# Identification of genes associated with platinum drug sensitivity and resistance in human ovarian cancer cells

**DOI:** 10.1038/sj.bjc.6602447

**Published:** 2005-02-22

**Authors:** D Roberts, J Schick, S Conway, S Biade, P B Laub, J P Stevenson, T C Hamilton, P J O'Dwyer, S W Johnson

**Affiliations:** 1 University of Pennsylvania Cancer Center, Philadelphia, PA, USA; 2 Department of Medical Oncology, Fox Chase Cancer Center, Philadelphia, PA, USA

**Keywords:** cisplatin, drug resistance, ovarian cancer, microarray, Stat1

## Abstract

Platinum-based chemotherapeutic regimens are ultimately unsuccessful due to intrinsic or acquired drug resistance. Understanding the molecular basis for platinum drug sensitivity/resistance is necessary for the development of new drugs and therapeutic regimens. In an effort to identify such determinants, we evaluated the expression of approximately 4000 genes using cDNA microarray screening in a panel of 14 unrelated human ovarian cancer cell lines derived from patients who were either untreated or treated with platinum-based chemotherapy. These data were analysed relative to the sensitivities of the cells to four platinum drugs (*cis*-diamminedichloroplatinum (cisplatin), carboplatin, DACH-(oxalato)platinum (II) (oxaliplatin) and *cis*-diamminedichloro (2-methylpyridine) platinum (II) (AMD473)) as well as the proliferation rate of the cells. Correlation analysis of the microarray data with respect to drug sensitivity and resistance revealed a significant association of Stat1 expression with decreased sensitivity to cisplatin (*r*=0.65) and AMD473 (*r*=0.76). These results were confirmed by quantitative RT–PCR and Western blot analyses. To study the functional significance of these findings, the full-length Stat1 cDNA was transfected into drug-sensitive A2780 human ovarian cancer cells. The resulting clones that exhibited increased Stat1 expression were three- to five-fold resistant to cisplatin and AMD473 as compared to the parental cells. The effect of inhibiting Jak/Stat signalling on platinum drug sensitivity was investigated using the Janus kinase inhibitor, AG490. Pretreatment of platinum-resistant cells with AG490 resulted in significant increased sensitivity to AMD473, but not to cisplatin or oxaliplatin. Overall, the results indicate that cDNA microarray analysis may be used successfully to identify determinants of drug sensitivity/resistance and future functional studies of other candidate genes from this database may lead to an increased understanding of the drug resistance phenotype.

Since its introduction into the clinic 30 years ago, *cis*-diamminedichloroplatinum (II) (cisplatin) has had a significant impact on the treatment of solid tumours. Alone or in combination with other chemotherapeutic drugs, cisplatin and its less toxic analogue, carboplatin, are curative for testicular cancer and also prolong survival in other tumours including those of the ovary, lung, bladder and head and neck (Johnson *et al*, 2001). Successful treatment of tumours with these drugs, however, is limited by the development of tumour cell resistance. This has fuelled a search for new platinum analogues that exhibit nonoverlapping cytotoxicity profiles in cisplatin/carboplatin-refractory tumours. The development of such analogues has been achieved primarily through modification or substitution of the diammine carrier ligands of the parent compound.

The DACH platinum compounds are a series of complexes containing a diaminocyclohexane carrier ligand (Connors *et al*, 1972; Kidani *et al*, 1976; Rixe *et al*, 1996). These compounds have a unique cytotoxicity profile as compared to cisplatin and exhibit collateral sensitivity in some cisplatin-resistant cell lines (Burchenal *et al*, 1980). The clinical development of DACH platinum drugs was initially hampered due to unacceptable toxicities; however, significant interest has been rekindled by the development of the less toxic analogue, oxaliplatin ((DACH-(oxalato)platinum (II)). Oxaliplatin has shown activity alone or in combination with 5-fluorouracil/leucovorin in colon cancer, a disease that was previously considered to be unresponsive to platinum drugs (Cvitkovic and Bekradda, 1999). In addition to oxaliplatin, another unique platinum analogue, *cis*-diamminedichloro(2-methylpyridine) platinum (II) (AMD473), has been developed in an effort to circumvent cisplatin resistance. AMD473 is a ‘sterically hindered’ platinum complex that was designed to react preferentially with nucleic acids over thiol-containing molecules such as glutathione (Kelland, 1999). Studies have shown that AMD473 exhibits activity against acquired cisplatin-resistant cell lines and is active when administered by oral or intraperitoneal routes in human ovarian cancer xenografts (Raynaud *et al*, 1997; Holford *et al* 1998).

Tumour cells may exhibit intrinsic platinum resistance or may acquire resistance following multiple cycles of platinum-based chemotherapy. Platinum resistance is considered multifactorial and includes mechanisms that limit the formation of platinum–DNA adducts and mechanisms that prevent cell death following drug-induced damage (Johnson *et al*, 1998). More specifically, these mechanisms include decreased drug uptake, increased drug inactivation, increased repair of platinum–DNA adducts and increased platinum–DNA damage tolerance. Some of these mechanisms may be specific to the type of platinum drug used, whereas others may be pleiotropic. As the molecular basis for platinum resistance remains largely undefined, the identification of the specific genes/pathways involved will hopefully lead to new strategies to treat platinum-refractory tumours or prevent resistance from emerging.

The development of drug resistance models has been instrumental to the identification of resistance mechanisms. These models have been established primarily by repeatedly exposing drug-sensitive cells to chemotherapeutic drugs *in vitro.* Although many putative drug resistance mechanisms have been identified and characterised in these types of models, their relevance to clinical drug resistance has been difficult to prove. Therefore, it is important to study resistance models derived from tumours in order to identify clinically relevant resistance mechanisms. One such model that we have extensively studied is a panel of unrelated human ovarian cancer cell lines derived from patients who were either untreated or treated with platinum-based chemotherapy. The cell lines of this panel exhibit a wide range of sensitivity to cisplatin (40-fold) and other chemotherapeutic drugs (Johnson *et al*, 1997). In the present study, we have used this panel of cell lines to identify candidate genes associated with sensitivity/resistance to four platinum drugs by cDNA microarray screening. Although semiquantitative, this technique can delineate patterns of gene expression and identify clusters of up or downregulated genes within a group of samples. Our results indicate that coupled with the appropriate validation steps, this method is useful for the identification of genes involved in chemoresistance.

## MATERIALS AND METHODS

### Cell culture

The human ovarian cancer cell lines used in this study were isolated from patients who were either untreated or treated with platinum-based chemotherapy (Table 1). The OAW42 cell line, originally described by Hill *et al* (1984), was obtained from the European Collection of Cell Cultures. Cells were maintained at 37°C in a humidified incubator containing 5% CO_2_ in RPMI 1640 medium (Life Technologies, Grand Island, NY, USA) supplemented with 10% (v v^−1^) foetal calf serum (Atlanta Biologicals, Atlanta, GA, USA), 100 *μ*g ml^−1^ streptomycin, 100 U ml^−1^ penicillin, 0.3 mg ml^−1^ glutamine and 0.25 U ml^−1^ insulin (porcine).

### Cytotoxicity assay

Cytotoxicity (IC_50_) values for the four platinum drugs were determined using the 3-(4,5-dimethyl-thizol-2-yl)-2,5-diphenyltetrazolium bromide (MTT) assay (Hansen *et al*, 1989). Cells (100–4000 well^−1^) were plated in 150 *μ*l of medium/well in 96-well microtitre plates. Following overnight incubation, cells were exposed to a range of drug concentrations. After 72 h, 40 *μ*l of 5 mg ml^−1^ MTT were added per well and the plates were incubated for 2 h at 37°C. The cells were then lysed by adding 100 *μ*l of 20% (w v^−1^) sodium dodecyl sulphate (SDS), 50% (v v^−1^) *N,N*-dimethylformamide (pH 4.7), and then incubating overnight at room temperature. The absorbance at 595 nm was determined using a Bio-Tek EL_X_800 microplate reader (Bio-Tek Instruments, Winooski, VT, USA). The reported values are the average of triplicate determinations made on at least two separate occasions. Relationships between the drugs were assessed by the Spearman's rank correlation test using ⩽0.05 as a significance threshold. For studies using tyrphostin B42 (AG490 – Calbiochem), cells were pretreated for 1 h with the inhibitor followed by the addition of platinum drugs as described above.

### Cell doubling time

Doubling time was determined using a similar assay as described by Ross *et al* (2000). Cells (150 *μ*l) were plated at two densities into six wells of a 96-well microtitre plate. At 24, 48 and 72 h time points, 40 *μ*l of 5 mg ml^−1^ MTT were added per well. After 2 h at 37°C, the cells were lysed by adding 100 *μ*l of 20% (w v^−1^) SDS, 50% (v v^−1^) *N,N*-dimethylformamide (pH 4.7), and then incubated overnight at room temperature. The absorbance of each well at 595 nm was recorded. Doubling times were calculated using the formula: *N*/*N*_0_=e^*kt*^, where *N* is the corresponding value at time zero. The constant *k* was calculated for each cell line between 24 and 72 h, the period of time in which the cell proliferation rate was maximal. The doubling time was then determined using the above formula with *N*/*N*_0_=2.

### RNA isolation

Total cellular RNA was extracted from the cell lines by a modification of the single-step method described by Chomczynski and Sacchi (Chomczynski and Sacchi, 1987; Chomczynski and Mackey, 1995) using guanidine isothiocyanate. RNA was precipitated from the aqueous phase by the addition of isopropanol. Following centrifugation, RNA pellets were washed with 75% ethanol, resuspended in DEPC-treated water and treated with DNase I. RNA integrity was assessed by ethidium bromide staining following agarose-gel electrophoresis and quantitated by absorbance at 260 nm. Samples were stored at −80°C under ethanol.

### Preparation of cDNA probes

Total RNA (2.0 *μ*g) was combined in a 1.5 ml microcentrifuge tube with 10 *μ*g of oligo-dT (Research Genetics) in a final volume of 10 *μ*l and incubated at 70°C for 10 min and placed on ice for 5 min. The cDNA synthesis reaction contained a mixture of 6.0 *μ*l of 5 × first strand Buffer (Life Technologies), 1.0 *μ*l 100 mM DTT, 1.5 *μ*l dA/dG/dT (10 mM each), 10 *μ*l [*α*^−33^P]dCTP (100 *μ*Ci), and 1.5 *μ*l MMLV reverse transcriptase (200 U *μ*l^−1^). Samples were incubated at 37°C for 1.5 h. Nucleotides and unincorporated ^33^P were removed by gel chromatography using Micro Bio-Spin 30 columns (Bio-Rad Laboratories, Hercules, CA, USA). The samples were denatured by incubating at 100°C for 3 min and placed on ice.

### cDNA microarray screening

Microarray analysis was carried out using the recommended procedure supplied by Research Genetics Inc. The Named Human Genes filters (GF211), which are spotted with 4132 cDNA elements, were prehybridized in 5 ml MicroHyb solution (Research Genetics) containing 5 *μ*g of poly-dA overnight at 42°C in a hybridisation oven (Model H010-1, Stovall Life Science Inc., Greensboro, NC, USA). The denatured probes were then added to this solution and incubated 14–18 h at 42°C. The membranes were washed briefly with 200 ml of wash buffer I (2 × SSC, 1% SDS) at room temperature, followed by two 20 min washes with the same buffer at 50°C, and two washes with 200 ml of wash buffer II (0.5 × SSC, 1% SDS) at 55°C for 20 min. The filters were removed, immediately wrapped in plastic and imaged on a Storm 840 phosphorimager (Molecular Dynamics, Sunnyvale, CA, USA), following a 5-day exposure to the imaging plate. For reuse, membranes were stripped by incubating for 20 min in 0.5% (w v^−1^) SDS that had been brought to 100°C and repeating. Stripped filters were reimaged following a 24 h exposure to insure complete removal of radioactivity.

### Data analysis

The data obtained from the phosphorimager was analysed using Pathways software (Research Genetics). This software utilises the raw image obtained from the phosphorimager in order to align the image and determine expression values for all of the elements. The raw data were downloaded into Microsoft Excel for final analysis. Also, a signal-to-noise ratio was obtained for all of the hybridised filters by dividing the mean of the raw data by the background value. Only the hybridisations that yielded signal-to-noise ratios above 1.5 were used in the subsequent data analysis. In order to correct for background, the average value of the lowest 1% of the genes expressed for each sample was subtracted from each raw data point to obtain a background corrected expression value. The data were then normalised by subtracting the mean and dividing by the standard deviation of the expression values for the entire data set for each cell line (global means method). RNA from each cell line was subject to microarray analysis at least twice. The normalised data obtained from duplicated hybridisations were averaged to obtain a final data set. Reproducibility of the data was assessed by calculating a percent error (s.d./mean × 100) for each gene element.

The data were cropped to a final set of 2000 elements by eliminating genes with relatively low expression and low standard deviation across the panel of cell lines. This was carried out by measuring the expression of 51 genes in each of the 14 cell lines by quantitative ‘real-time’ PCR. We considered a gene ‘validatable’ if the Spearman and/or Pearson correlation coefficient was ⩾0.6 for the microarray/PCR data. A factor (*P*) was calculated by multiplying the average of the expression of each gene for all the cell lines with the standard deviation. Genes with *P*-values below 100 000 could not be consistently validated by RT–PCR and were eliminated. We also eliminated genes in which the median expression value across the cell lines was low (see Web Supplement for more detail).

The identification of candidate drug sensitivity/resistance genes was carried out by several statistical methods. Correlation coefficients (Pearson and Spearman) were calculated for the expression of each gene relative to the proliferation rate and platinum drug sensitivity for the entire panel of cell lines. The correlation coefficients were then ranked from highest to lowest and *vice versa* in order to obtain a list of genes associated with proliferation and/or platinum drug sensitivity. Hierarchical clustering was carried out using the Cluster and TreeView programs developed by Eisen *et al* (1998). Data for each gene element were normalised and mean centred using the software.

### Quantitative RT–PCR

Validation of the expression of candidate genes identified by microarray analysis was carried out by ‘real-time’ quantitative PCR using a Roche LightCycler with SYBR green chemistry. Total RNA (2 *μ*g) from each sample was reverse transcribed using the Superscript II First Strand cDNA synthesis kit (Invitrogen Inc., Huntsville, AL, USA). Reaction conditions were optimised for each primer set. Measurements were made from each cDNA reaction in duplicate and normalised based on the average of the normalised expression of four housekeeping genes. The housekeeping genes were chosen using the microarray data and selection was limited to the genes that exhibited the low variability in expression across the panel of ovarian cancer cell lines. Primer sequences and reaction conditions are listed at www.realtimeprimers.org.

### Western blot analysis

Cell extracts were prepared by lysing cells grown in 10 cm culture dishes with 50 mM Tris-HCl, pH 7.4, containing 1% NP-40, 0.25% sodium deoxycholate, 150 mM NaCl, 1 mM EDTA, 1 mM PMSF, 1 *μ*g ml^−1^ protease inhibitors, 1 mM NaF and 1 mM Na_3_VO_4_. Protein concentrations were determined using the Bradford protein assay and equal amounts of extract (15 *μ*g) were resolved on 10% SDS–polyacrylamide gels. Following transfer to nylon membranes, the blots were blocked overnight at 4°C in Tris-buffered saline (TBS) containing 0.1% Tween-20 detergent and 5% nonfat dry milk. Membranes were then incubated for 1 h at room temperature with a 1 : 2000 dilution of monoclonal mouse anti-Stat1 antibody (Santa Cruz, Santa Cruz, CA, USA). Following three washes in TBS-T, the membranes were incubated in 1 : 3000 dilution of horseradish peroxidase-linked anti-mouse secondary antibody (Santa Cruz, Santa Cruz, CA, USA). Following several washes in TBS-T, the Stat1 protein bands were visualised by chemiluminescence (ECL, Amersham Biosciences, Piscataway, NJ, USA). Individual protein bands were quantitated by densitometry.

### Transfections

The full-length Stat1-pcDNA3.1 GeneStorm expression ready plasmid was purchased from Invitrogen Inc. (Huntsville, AL, USA). The plasmid was transfected into A2780 cells using Lipofectamine and selected with zeocin. Individual clones were isolated, propagated and examined for Stat1 expression by Western blotting and for drug sensitivity using the MTT assay.

## RESULTS

The human ovarian cancer cell lines used in this study are listed in Table 1. These cell lines were established from patients who were either untreated or treated with platinum-based chemotherapy. Seven of these cell lines represent patients who failed chemotherapy. The others are derived from chemotherapy-naïve patients; however, several of these (A1847, SKOV3, OVCAR5, OVCAR7) exhibit intrinsic platinum resistance. The only two related cell lines are PEO1 and PEO4. The latter was established from the same patient as PEO1; however, this was carried out after the patient developed resistance to platinum-based chemotherapy. Cell doubling times and platinum drug sensitivities were measured in this cell line panel using an MTT assay (Table 2). The range in proliferation rate was approximately three-fold with A2780 having the shortest doubling time (13.5 h) and OVCAR7 the longest (44.7 h). A wide range of sensitivity to the four platinum drugs was also observed. As shown in Table 3, a strong similarity was observed between the cytotoxicity profiles of cisplatin and carboplatin (*r*=0.95), whereas oxaliplatin did not show a high correlation with the sensitivity pattern of these two drugs (*r*=0.37 and 0.35, respectively). Although not as significant, there was an association between cisplatin and carboplatin sensitivities with that of AMD473 (*r*=0.62 and 0.60, respectively). Significant similarities were also observed between oxaliplatin and AMD473 (*r*=0.74). Decreased cell doubling time was associated with resistance to oxaliplatin (*r*=0.64) and AMD473 (*r*=0.60), but not to the other two platinum drugs.

In order to identify genes and gene expression profiles associated with cell doubling time and platinum drug sensitivity, we measured the constitutive expression of approximately 4000 genes in the 14 human ovarian cancer cell lines using cDNA microarray screening. RNA from each cell line was ^33^P-labelled and hybridised to Research Genetics GF211 Named Human Genes filters. Duplicate hybridisations were performed for each RNA sample on different days using a different filter from the same manufactured lot. Following background correction and global means normalisation, variability was assessed for replicates by determining a percent error associated with each gene element. Of the 4132 elements examined, the majority of expression values (66%) were associated with less than 35% error between duplicates, 25% were associated with 25 to 50% error and 9% were associated with greater than 50%. An important issue in analysing microarray data is to determine which gene expression values are most likely to be real or ‘validatable’. This problem was approached by using quantitative RT–PCR measurements for 51 genes to guide the processing of the microarray data. We considered a gene validatable if the Pearson or Spearman correlation coefficient derived between the RT–PCR and microarray data was 0.6 or higher. Using the microarray data, a factor was calculated based on the variability of the gene expression data and the mean intensity of each gene across the panel of cell lines. This was used to extract a data set that represented the genes that had a higher probability of being validated by RT–PCR. Based on this process, we estimated that approximately 70% of the genes in the data set were ‘validatable’. The Web Supplement contains a full list of the genes measured along with the correlation coefficients calculated for the cell doubling times and platinum drug sensitivities. The correlation between the RT–PCR and microarray results are also provided.

Resistance to platinum drugs is multifactorial, thus it is unlikely that the expression of a single gene would be strongly associated with platinum drug sensitivity/resistance in a panel of unrelated cell lines. We analysed our data set with the expectation that candidate genes would emerge with lower correlation coefficients and higher false discovery rates. Another obstacle to selecting causal sensitivity/resistance genes is that some gene expression changes may serve as markers of resistance, but not contributing functionally to the phenotype. Despite these potential limitations, we proceeded with the identification of genes associated with cell proliferation and platinum drug sensitivity was carried out using several methods including (1) correlation with respect to proliferation rate and drug sensitivity, (2) hierarchical clustering to identify patterns of gene expression in the whole panel of cell lines and (3) identifying differentially expressed genes in individual cell lines. Spearman rank correlation coefficients were calculated for each gene *vs* proliferation rate and platinum drug sensitivity in the 14 cell lines and the genes were ranked based on positive or negative correlation. Using a correlation coefficient of 0.5 or greater as a threshold, 47, 55 and 90 genes were associated with decreased sensitivity to cisplatin, oxaliplatin and AMD473, respectively. Table 4 lists genes that are most associated with resistance to these three platinum drugs. The remainder of the list as well as the entire data set is available in the Web Supplement. Of note, increased Stat1 expression was associated with decreased cisplatin and AMD473 sensitivity. Increased expression of chromatin assembly factor-I and syndecan 4 was associated with decreased sensitivity to both oxaliplatin and AMD473. Using a correlation coefficient of −0.5 or less as a threshold, 38, 56 and 104 genes were associated with decreased sensitivity to cisplatin, oxaliplatin and AMD473, respectively. From these analyses, we selected a number of candidates for validation by quantitative RT–PCR. Table 5 lists a set of 23 such genes along with the Spearman rank correlation coefficients derived with respect to platinum drug sensitivity. The correlation between expression values obtained by cDNA microarray analysis and quantitative RT–PCR is also shown.

**Figure 1:**
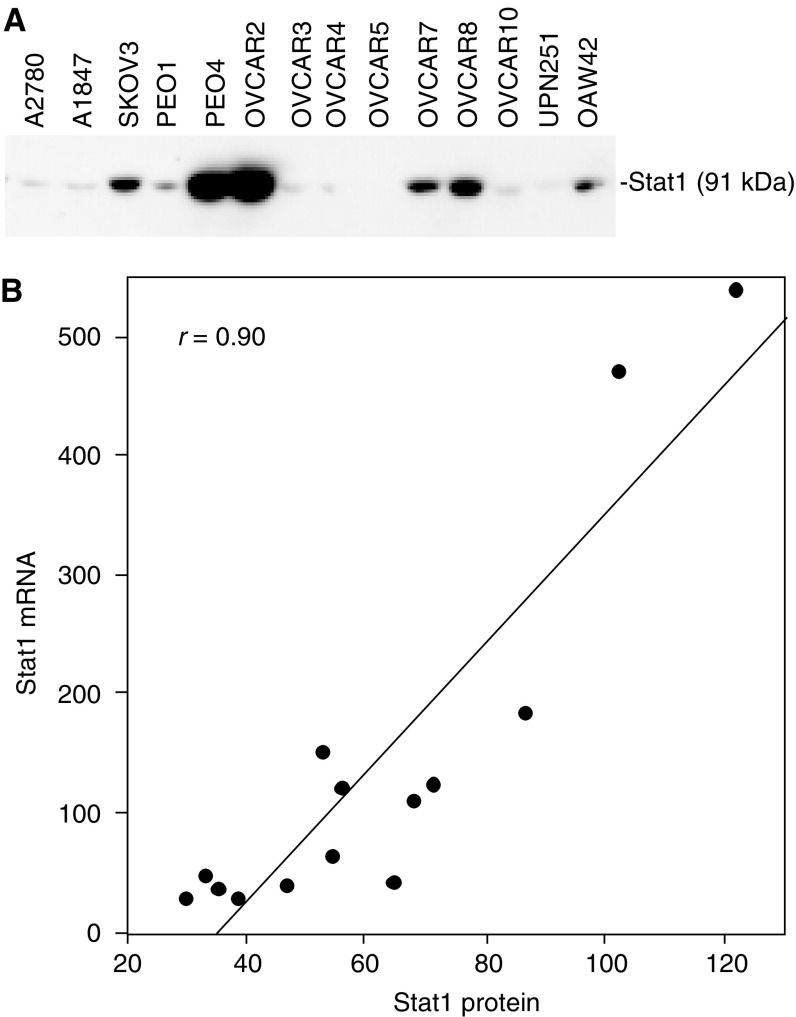
Expression of Stat1 protein in a panel of 14 human ovarian cancer cell lines. (**A**) Equal amounts (25 *μ*g) of whole-cell extract were resolved by SDS–PAGE and analysed by Western blot analysis using anti-Stat1 antibody; (**B**) comparison of the data obtained by Western blot analysis and quantitative ‘real-time’ RT–PCR. Measurements were made from each cDNA reaction in duplicate and normalised based on the average of the normalized expression of four housekeeping genes.

**Figure 2:**
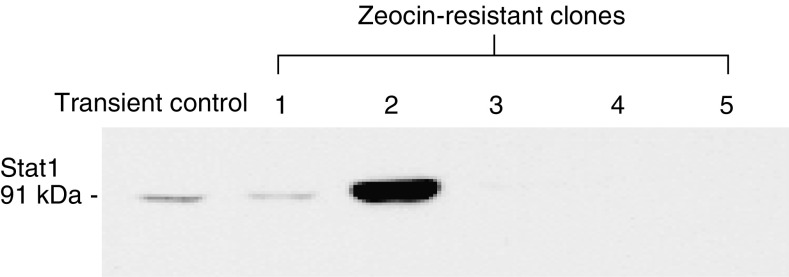
Isolation of Stat1-expressing clones. The full-length Stat1 cDNA was transfected into A2780 cells. Zeocin-resistant clones were isolated, propagated and assessed for Stat1 expression by Western blot analysis. As a positive control, A2780 cells were transiently transfected with the same Stat1 cDNA construct.

**Figure 3:**
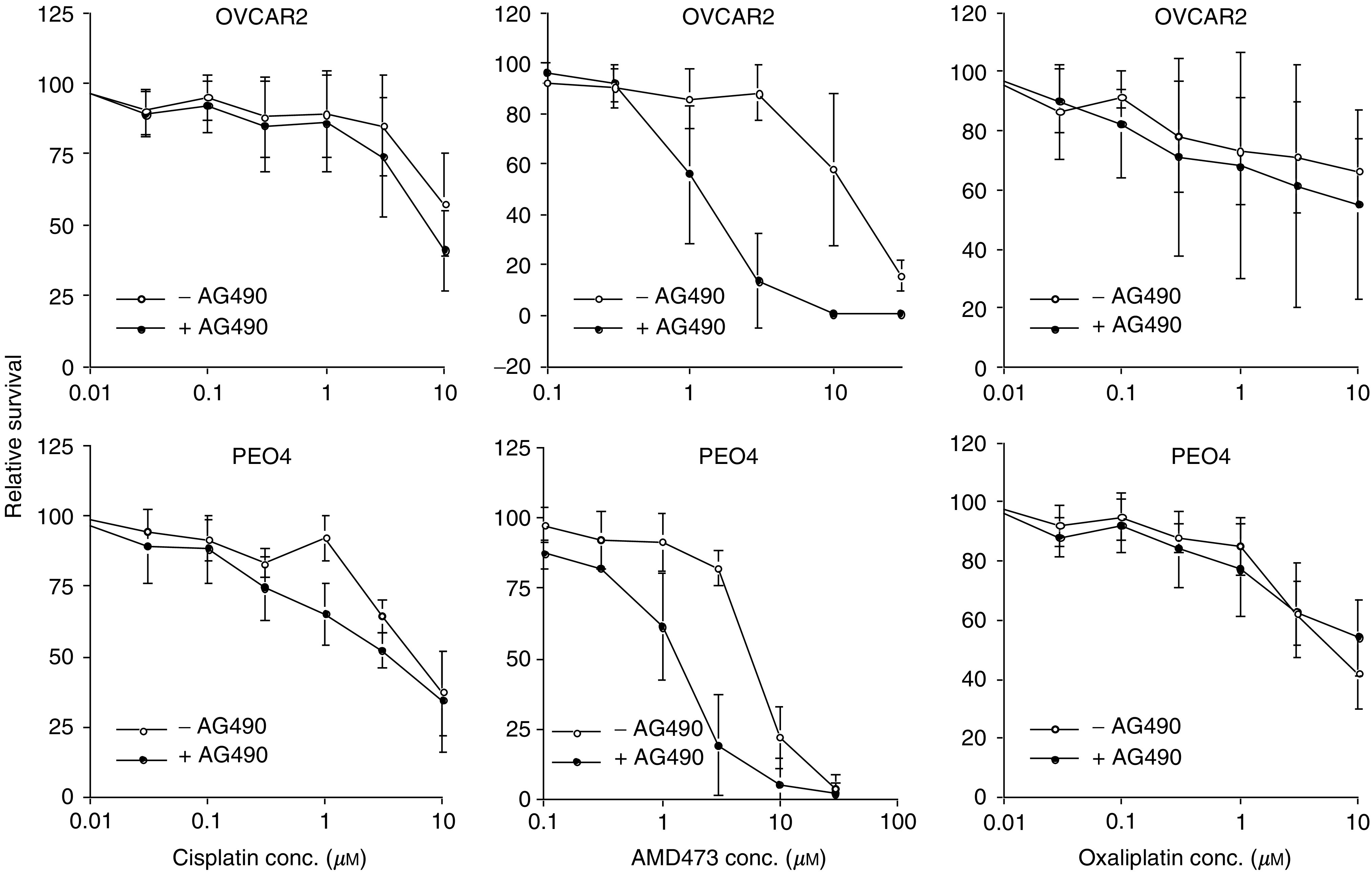
Effect of AG490 on platinum drug sensitivity in OVCAR2 and PEO4 cells. Cells were pretreated with 50 *μ*M AG490 for 1 h followed by exposure to a range of platinum drug concentrations. Survival was determined after 72 h using the MTT reagent. The results are the average of triplicate measurements obtained on at least two separate occasions. The error bars represent the standard deviation of each data point.

From the microarray data, it was evident that constitutive Stat1 expression was associated with both decreased cisplatin and AMD473 sensitivity in the panel of cell lines (*r*=0.65 and 0.76, respectively). This was confirmed by quantitative RT–PCR (Table 5). Stat1 protein levels were measured by Western blot analysis of protein extracts prepared from the same set of cell lines (Figure 1). The highest level of Stat1 expression was observed in PEO4 and OVCAR2 cells. Quantitative analysis of the blot revealed a high correlation between Stat1 protein levels and the expression data obtained by RT–PCR (*r*=0.94). The full-length cDNA for Stat1 was stably transfected into the multidrug-sensitive A2780 cell line. Five zeocin-resistant colonies were propagated and assessed for constitutive Stat1 expression by Western blot analysis (Figure 2). Two of the five stable transfectants contained elevated Stat1 levels, while the other three served as controls as no significant increase in Stat1 expression was observed relative to the parental cells. This is likely due to partial integration of the plasmid into the host genome resulting in the expression of only the neomycin resistance gene. The sensitivity of the clones to cisplatin, AMD473 and oxaliplatin was measured using an MTT assay (Table 6). The two Stat1-expressing clones exhibited decreased sensitivity to cisplatin and AMD473, but not change in sensitivity to oxaliplatin. Following several passages of the clones, Stat1 expression decreased and the sensitivity of the cells to cisplatin and AMD473 was similar to that of A2780 (data not shown). This could be due to the existence of a small population of cells within the colony exhibiting decreased Stat1 expression. Since Stat1 causes reduced cell proliferation, these cells may be enriched in the overall cell population after multiple passages. Pharmacologic inhibition of Jak/Stat signalling was studied using the Jak2 inhibitor, AG490 (Figure 3). The optimal concentration of AG490 required to inhibit Stat1 phosphorylation was determined by Western blot analysis (data not shown). OVCAR2 and PEO4 cells were pretreated with 50 *μ*M AG490 followed by treatment with a range of platinum concentrations. AMD473 sensitivity was enhanced 4.1-fold in PEO4 cells (*P*=0.001) and 9.1-fold in OVCAR2 cells (*P*=0.042) in the presence of AG490. Although a 1.3- and 1.8-fold increase in cisplatin sensitivity was observed in PEO4 and OVCAR2 cells, respectively, this effect was not statistically significant. There was no significant change in the sensitivity of either cell line to oxaliplatin following AG490 exposure.

## DISCUSSION

In order to identify molecular determinants of chemotherapeutic drug sensitivity and resistance, one must establish a clinically relevant model. Several groups have produced drug-resistant cancer cell lines; however, most of these have been derived artificially by exposing sensitive cells repeatedly to increasing concentrations of a chemotherapeutic drug. Although such models have their own merits, we have approached the problem of resistance by studying a panel of unrelated tumour cell lines. These cell lines not only exhibit a wide range of platinum drug sensitivity but were also established from the tumours of ovarian cancer patients who were either untreated or treated with platinum-based chemotherapy. In a previous study (Johnson *et al*, 1997), we defined the cellular pharmacology of cisplatin in these cell lines and discovered that platinum–DNA damage tolerance is a major mechanism of cisplatin resistance. To determine the molecular basis for this potentially clinically relevant resistance mechanism, we applied cDNA microarray analysis to the same set of cell lines in the present study. One limitation to our model is the overall number of cell lines examined relative to the large amount of expression data obtained. This significantly increases the number of genes that are found to be associated with the sensitivity/resistance phenotype. Another potential limitation is the selection of the MTT assay to measure platinum sensitivity. This assay is easy to conduct and provides data rapidly; however, the data may be subject to some variability when compared to that obtained by other cytotoxicity assays. In support of our approach, however, Perez *et al* (1993) demonstrated previously that cisplatin sensitivity as measured by the MTT assay correlates well with that of the sulphorhodamine B and clonogenic assays.

The variability observed in cytotoxicity profiles between platinum drugs is due, in part, to the composition of their carrier ligands. This ultimately affects the structure and conformation of the resulting platinum–DNA adducts. Although cisplatin and carboplatin contain different leaving groups, the structure of their reactive species is the same. Thus, both drugs show very similar cytotoxicity profiles. Oxaliplatin exhibited a unique cytotoxicity profile compared to that of cisplatin and carboplatin, which is consistent to that observed by others (Burchenal *et al*, 1980; Rixe *et al*, 1996). We also examined the sensitivity of the cell lines to AMD473, a platinum complex that has been shown previously to circumvent the acquired cisplatin resistance phenotypes in some *in vitro* models (Raynaud *et al*, 1997). Crossresistance of the cells to AMD473 was observed for some of the cell lines, however, the correlation coefficients obtained between AMD473 and the other three platinum drugs indicate that the structure of this complex imparts somewhat unique activity. We have shown previously that many of the cisplatin-resistant cell lines in our panel exhibit significant crossresistance to nonplatinum chemotherapeutic agents (Johnson *et al*, 1997). This suggests that tolerance or antiapoptotic mechanisms may be responsible for resistance in these cells resulting in decreased sensitivity to structurally unrelated drugs.

One can approach the problem of identifying resistance genes using several strategies. First, correlation coefficients can be used to find genes that are positively and negatively associated with chemoresistance across the entire panel of cell lines. Secondly, one can use supervised or unsupervised methods such as hierarchical clustering to identify gene expression patterns associated with a subset of resistant cells. Finally, one can identify genes that are significantly up- or downregulated in individual cells relative to all of the cell lines. The most effective strategy may be to use a variety of statistical approaches for gene discovery. Validating gene expression is also critical to the identification of candidate genes for functional studies. For expression profiling studies, quantitative ‘real-time’ RT–PCR offers a rapid and sensitive method for this step. Another important use of quantitative RT–PCR data is to facilitate the processing of microarray data to include only the set of genes that are considered ‘validatable’. Genes that are highly expressed and that exhibit high variability in expression have a high probability of correlating positively with RT–PCR data. In contrast, microarray data that represents low variability and low levels of gene expression will correlate poorly with RT–PCR data. In this study, we used RT–PCR data to guide the processing of our microarray data in order to obtain a set of ‘validatable’ genes. This is likely to be the first report that describes such an approach for cleaning a set of semiquantitative array data.

We focused our functional studies on the Jak/Stat signalling pathway for several reasons. A positive correlation was observed between Stat1 expression and decreased sensitivity to both cisplatin and AMD473. The relatively high correlation coefficients obtained were due, in part, to a higher expression of Stat1 in both the OVCAR2 and PEO4 cell lines. In addition, the PEO4 cell line contained significantly higher levels of Stat1 mRNA (15-fold) and protein (six-fold) as compared to its drug-sensitive counterpart, PEO1. We also observed a positive correlation between the expression of interferon-stimulated gene factor 3 (ISGF3)*γ* (IRF9, p48), a transcription factor subunit that forms a heterodimer with Stat1, and decreased sensitivity to cisplatin (*r*=0.70) and AMD473 (*r*=0.40). Interestingly, the PEO4 cell line also contained elevated expression levels of other interferon-inducible genes when compared to the other cell lines. These included interferon alpha-induced 11.5 kDa protein, interferon-inducible protein 9–27, interferon-inducible protein p78, interferon-induced 17 kDa protein, interferon-inducible protein 1–8D, interferon-induced 56 kDa protein and interferon-inducible protein 1–8U. The upregulation of these genes may be due, in part, to elevated levels of the ISGF3 transcription factor complex. In fact, we evaluated the constitutive activity of this complex using an ISRE element linked to a luciferase reporter gene and found that PEO4 cells exhibit a two-fold higher level of ISRE transcriptional activity as compared to platinum-sensitive PEO1 cells (data not shown).

The Jak/Stat pathway represents a major signalling pathway that enables a cell to interpret and react to inflammatory signals and cytokines (Leonard and O’Shea, 1998). This pathway utilises cytosolic transcription factors known as Stat's (signal transducer and activator of transcription) to transduce an extracellular signal to the nucleus. ISGF3 is a multisubunit transcription factor required for transcriptional activation of interferon-stimulated genes in response to interferons and other cytokines. The ISGF3 complex contains two major components: ISGF3*γ* and ISGF3*α*, the latter comprised of two Stat proteins (Stat1*α*, Stat1*β* or Stat2). Treatment of cells with interferons or cytokines leads to receptor-mediated signal transduction and tyrosine phosphorylation of the Stat subunits by Janus kinases (Jak1, Jak2, Jak3 or Tyk2). The phosphorylated complex is subsequently translocated to the nucleus for target gene activation. A role for these transcription factors in resistance to platinum drugs has not been reported previously; however, a report by Luker *et al* (2001) showed that overexpression of ISGF3 (IRF9) confers resistance to paclitaxel. In their study, paclitaxel resistance was not observed following transfection of Stat1 or Stat2 cDNAs. Our functional studies demonstrated that Stat1 overexpression confers resistance to cisplatin and AMD473, but not to oxaliplatin. Conversely, inhibition of Jak/Stat signaling using the Jak2 inhibitor, AG490, resulted in increased sensitivity to cisplatin and AMD473, but not to oxaliplatin. Our previous study indicated that PEO4 and OVCAR2 cells have a relatively high level of platinum–DNA damage tolerance as compared to the other cell lines in this panel (Johnson *et al*, 1997); thus, we suspect that Jak/Stat signalling may be involved in preventing cell death following DNA damage. This may occur indirectly through the induction of antiapoptotic genes or by reducing cell proliferation, thus enabling more time to repair DNA lesions. We observed that some cell lines exhibiting increased platinum drug resistance do not contain elevated levels of Stat1. For example, OVCAR10 is 50- and 12-fold resistant to cisplatin and AMD473, respectively, relative to A2780 cells and shows a high level of platinum–DNA damage tolerance (Johnson *et al*, 1997). This is not unexpected since our study included a panel of unrelated cell lines, and given the fact that platinum resistance is multifactorial, a number of other potential mechanisms could be responsible.

Other investigators have reported an effect of AG490 on the survival of various types of cancer including breast (Burke *et al*, 2001; Garcia *et al*, 2001), leukaemia (Spiekermann *et al*, 2001), myeloma (De Vos *et al*, 2000), ovarian (Burke *et al*, 2001; Arbel *et al*, 2003), pancreatic (Toyonaga *et al*, 2003) and prostate (Ni *et al*, 2000). Since Jak2 is capable of phosphorylating a number of downstream targets such as Stat1, Stat3 and Stat5, the effect of AG490 on platinum sensitivity in our model may be the result of inhibiting multiple survival signalling pathways. Interestingly, we did not observe increased sensitivity to nonplatinum chemotherapeutic drugs when combined with AG490 (data not shown). We also found that cells that do not express relatively high levels of Stat1 such as A2780 or PEO1 cells are not sensitised to cisplatin or AMD473 in the presence of AG490. This suggests that, although such inhibitors may be cytotoxic as single agents, increased therapeutic benefit may not be observed when combined with platinum drugs in tumours that do not exhibit increased Jak/Stat signalling. Thus, identifying the subset of patients whose tumours exhibit upregulation of this pathway could be useful for combined therapy with a platinum drug. This observation emphasises the importance of tailoring treatment to the expression profile of a particular tumour. In addition to Stat1, we selected a number of other genes for validation experiments based, in part, on their association with platinum drug sensitivity (Table 5). Among these are included extracellular matrix proteins, cytokines, growth factors, interferon-inducible genes and calcium binding proteins. Currently, there are no specific studies to suggest these genes are involved in cisplatin resistance; however, one may speculate a role for them in prosurvival signalling based on their putative functions.

Similar to the resistance phenotype, sensitivity to platinum drugs may be conferred by increased expression of a variety of genes. Our analysis of oxaliplatin and AMD473 sensitivity revealed a common theme, which was the expression of a considerable number of transcripts involved in RNA transport and processing. These included the heterogeneous ribonucleoproteins A/B, H, A1, A2/B1, R, C, hPrp4 and several pre-mRNA splicing factors. A role for these genes in conferring platinum sensitivity is unknown; however, one theory is that they may create alternatively spliced forms of some mRNA species, thus altering their function. There are reports of this phenomenon in the literature. Alternatively spliced genes have been discovered that confer opposing apoptotic effects including variants of survivin, Fas receptor and CC3, a metastasis suppressor (Conway *et al*, 2000; Jenkins *et al*, 2000; Whitman *et al*, 2000). It is unlikely that cDNA microarray analysis can identify splice variants since the technique relies on hybridisation. However, the observed differential expression of genes involved in RNA processing suggests that analysing ovarian cancer cells for alternatively spliced transcripts may reveal novel mechanisms of platinum drug sensitivity.

In recent years, a number of reports have provided gene expression profiles associated with intrinsic and acquired drug resistance (Scherf *et al*, 2000; Dan *et al*, 2002; Higuchi *et al*, 2003). These studies were carried out using various microarray platforms and model systems. Nonetheless, we compared our data to that produced from other studies to find platinum sensitivity and resistance genes. The gene expression database established by Scherf *et al* (2000) utilised growth inhibition data for the NCI60 cell lines along with expression measurements for 1376 genes. This panel of cell lines contained only six ovarian cancer cell lines, so instead of comparing our data to this small subset of cells, we compared data for the entire panel. We did not observe commonality in the two data sets for genes associated with cisplatin sensitivity; however, the expression of three genes (ISGF3*γ*, MSSP-1 and transforming growth factor (TGF)-beta.) was found to be associated with cisplatin resistance. In the present study, we provide correlative evidence for ISGF3*γ* expression and decreased cisplatin sensitivity. There are no reports implicating MSSP-1 (RBMS1, RNA binding motif, single-stranded interacting protein) in cisplatin resistance. MSSP-1 possesses versatile functions including stimulation of DNA replication, transcriptional regulation, apoptosis induction and cell transformation coordinated by c-Myc (Niki *et al*, 2000). TGF-beta is a multifunctional peptide that controls proliferation, differentiation and other functions in many cell types. Teicher *et al* (1997) examined the role of TGF-beta in multidrug resistance by transfecting TGF-beta into murine mammary carcinoma cells. In monolayer culture, the TGF-beta-expressing cells were not resistant to cisplatin relative to control cells, but were markedly resistant when grown as solid tumours in mice. We did not find similarities in our set of putative cisplatin resistance genes when compared to the data provided in two other studies. Dan *et al* (2002) examined the expression of 9216 genes in 39 human cancer cell lines in relation to growth inhibition data for 55 anticancer drugs. The aldo-keto reductase gene and damage-specific DNA binding protein 2 were associated with sensitivity to at least 20 of the drugs including cisplatin and expression of the LIM domain kinase 2 gene was associated with resistance. Higuchi *et al* (2003) approached the problem of identifying cisplatin resistance genes using acquired cisplatin resistance head and neck cancer cells. This group reported alterations in the expression of a glycoprotein hormone and membrane proteins in this model. These results underscore not only the potential complexity of platinum drug sensitivity and resistance but also demonstrates the heterogeneity in results that are obtained from different cell models and microarray platforms.

In summary, high-throughput technologies such as cDNA microarray analysis have facilitated the discovery of the molecular determinants of chemotherapeutic drug sensitivity/resistance. However, expression profiling is a relatively new tool, thus its potential value for drug target discovery is unclear at this early stage. This will become increasingly apparent as the number of pharmacogenomic databases increase and comparisons can be made across different platforms and cellular models. Our study provides a significant amount of gene expression data that may be useful for other investigators interested in chemotherapeutic drug resistance. The results of our study also emphasise the importance of validating results obtained by cDNA microarray analyses. From these data, we have shown that not only is increased Stat1 expression associated with resistance to some platinum drugs but also the Jak/Stat signalling pathway may be a useful target for the design of inhibitors. Although not in the scope of the present study, functional studies of other genes in this data set may lead to the identification of potential targets for enhancing platinum drug sensitivity.

## Figures and Tables

**Table 1:** Human ovarian cancer cell lines used in this study

**Cell line**	**Phenotype**
A2780	Untreated ovarian tumour
A1847	Untreated ovarian tumour
SKOV3	Ovarian tumour, cisplatin sensitive
PEO1	Ovarian tumour, cisplatin sensitive
PEO4	Ovarian tumour (PEO1) after patient became refractory
OVCAR2	Ovarian tumour from a cisplatin-refractory patient
OVCAR3	Ovarian tumour from a cisplatin-refractory patient
OVCAR4	Ovarian tumour from a cisplatin-refractory patient
OVCAR5	Untreated ovarian tumour
OVCAR7	Untreated ovarian tumour
OVCAR8	Ovarian tumour from a platinum-refractory patient
OVCAR10	Ovarian tumour from a platinum-refractory patient
UPN251	Ovarian tumour from a platinum/paclitaxel-refractory patient
OAW42	Ovarian tumour, cisplatin sensitive

**Table 2:** Doubling times and platinum drug sensitivities of human ovarian cancer cell lines

		**Platinum drug sensitivity (*μ*M) (resistance relative to A2780)**			
**Cell line**	**Doubling time (h)**	**Cisplatin**	**Carboplatin**	**Oxaliplatin**	**AMD473**
A2780	13.5±3.2 (1.0)	0.4±0.1 (1.0)	6.8±3.0 (1.0)	0.03±0.01 (1.0)	1.1±0.2 (1.0)
A1847	26.6±0.6 (2.0)	4.4±1.1 (12.4)	88.3±21.0 (13.1)	0.18±0.04 (7.1)	11.8±3.2 (10.3)
SKOV3	32.4±3.6 (2.4)	6.7±2.1 (18.8)	96.8±4.6 (14.3)	4.75±0.64 (186.3)	18.0±1.4 (15.7)
PEO1	25.4±6.4 (1.9)	1.0±1.2 (2.7)	13.6±3.9 (2.0)	0.34±0.06 (13.3)	3.9±0.1 (3.4)
PEO4	24.1±5.2 (1.8)	9.1±0.2 (25.4)	120.5±24.7 (17.9)	3.75±0.85 (147.1)	18.9±0.8 (16.5)
OVCAR2	43.5±8.5 (3.2)	9.3±0.6 (25.9)	151.0±43.8 (22.4)	5.33±1.17 (208.8)	33.0±7.8 (28.8)
OVCAR3	22.1±0.5 (1.6)	4.6±1.1 (12.8)	48.3±6.7 (7.1)	5.33±2.30 (208.8)	16.8±1.8 (14.6)
OVCAR4	39.1±1.9 (2.9)	1.6±0.0 (4.5)	19.0±1.4 (2.8)	0.24±0.02 (9.2)	16.8±3.5 (14.7)
OVCAR5	17.5±1.1 (1.3)	2.4±0.6 (6.6)	35.0±4.2 (5.2)	0.23±0.08 (9.0)	16.9±1.6 (14.8)
OVCAR7	44.6±12.5 (3.3)	2.3±0.2 (6.4)	50.8±22.4 (7.5)	9.83±5.03 (385.6)	22.8±11.2 (19.9)
OVCAR8	25.0±2.6 (1.9)	6.8±0.1 (19.1)	67.2±28.5 (9.9)	0.26±0.06 (10.0)	21.6±0.4 (18.8)
OVCAR10	15.3±1.7 (1.1)	17.9±1.6 (50.0)	173.3±22.5 (25.7)	0.11±0.03 (4.3)	13.4±1.2 (11.7)
UPN251	22.9±4.9 (1.7)	5.6±0.8 (15.7)	63.0±12.7 (9.3)	3.18±0.25 (124.5)	17.7±1.2 (15.4)
OAW42	18.2±1.4 (1.4)	0.9±0.3 (2.4)	13.3±2.9 (2.0)	0.05±0.0 (1.8)	3.0±0.9 (2.6)

**Table 3:** Correlation coefficients^a^ derived from the relationships between the sensitivities of four platinum drugs in 14 human ovarian cancer cell lines

**Platinum drug**	**Cisplatin**	**Carboplatin**	**Oxaliplatin**	**AMD473**
Carboplatin	*r*=0.95, *P*=0.000	—	—	—
Oxaliplatin	*r*=0.37, *P*=0.198	*r*=0.35, *P*=0.215	—	—
AMD473	*r*=0.62, *P*=0.019	*r*=0.60, *P*=0.025	*r*=0.74, *P*=0.003	—
Doubling time	*r*=0.12, *P*=0.682	*r*=0.27, *P*=0.351	*r*=0.64, *P*=0.015	*r*=0.60, *P*=0.022

a Spearman's rank test was used to calculate correlation coefficients and *P*-values.

**Table 4:** Genes associated with resistance to cisplatin, AMD473 and oxaliplatin as measured by cDNA microarray analysis

**Unigene #**	**Accession #**	**Genes associated with cisplatin resistance**	***r* a**
Hs.20225	AA485750	Tuftelin interacting protein 11	0.749
Hs.444058	AA598621	Signal recognition particle receptor (‘docking protein’)	0.745
Hs.430541	AA431849	SON DNA binding protein	0.741
Hs.1706	AA291389	Transcriptional regulator ISGF3 gamma subunit	0.714
Hs.254321	T65118	Alpha-catenin	0.692
Hs.274485	AA464246	Major histocompatibility complex, class I, C	0.679
Hs.83469	AA496576	Transcription factor 11 (basic leucine zipper type)	0.670
Hs.458414	AA419251	Interferon-inducible protein 9–27	0.666
Hs.21486	AA488075	Signal transducer and activator of transcription 1	0.653
Hs.92004	R52541	EST	0.622
			
**Unigene #**	**Accession #**	**Genes associated with AMD473 resistance**	***r***
Hs.470489	AA427561	Heparan sulphate proteoglycan (HSPG2) mRNA	0.881
Hs.8136	AA680300	Endothelial PAS domain protein 1 (EPAS1)	0.763
Hs.21486	AA488075	Signal transducer and activator of transcription 1	0.763
Hs.75238	AA426096	Chromatin assembly factor-I p60 subunit	0.754
Hs.504789	AA148737	Syndecan 4 (amphiglycan, ryudocan)	0.741
Hs.204238	AA400973	Neutrophil gelatinase-associated lipocalin precursor	0.736
Hs.433326	H79047	Insulin-like growth factor binding protein 2	0.736
Hs.435238	AA460827	Protein phosphatase I inhibitor	0.714
Hs.355214	H44051	Keratin, type I cytoskeletal 14	0.697
Hs.18799	R73545	Flotillin 2	0.697
			
**Unigene #**	**Accession #**	**Genes associated with oxaliplatin resistance**	***r***
Hs.504789	AA148737	Syndecan 4 (amphiglycan, ryudocan)	0.859
Hs.437313	H11003	Endothelin 1 (alternative products)	0.742
Hs.80395	AA227885	Mal, T-cell differentiation protein	0.733
Hs.83577	AA195959	LIM protein MLP mRNA	0.728
Hs.194673	AA293653	Homolog of mouse MAT-1 oncogene mRNA	0.701
Hs.410104	H43049	Serine/threonine-protein kinase receptor R3 precursor	0.701
Hs.470843	AA039370	Transcriptional enhancer factor TEF-1	0.692
Hs.75238	AA426096	Chromatin assembly factor-I p60 subunit mRNA	0.688
Hs.75216	AA598513	Protein tyrosine phosphatase, receptor type, f polypeptide	0.679
Hs.251754	AA683520	Antileukoproteinase 1 precursor	0.674

The top 10 genes associated with decreased sensitivity to each drug is listed. The full set of genes is available in the Web Supplement.

a The Spearman's rank correlation coefficient calculated for each gene and each platinum drug is listed.

**Table 5:** Results of real-time quantitative RT–PCR analysis of gene expression associated with resistance to platinum drugs^a^. Data for the remainder of the genes analysed are available in the Web Supplement

**Gene**	**Cisplatin**	**Oxaliplatin**	**AMD473**	**Microarray/PCR correlation**
Signal transducer and activator of transcription 1	0.69	0.53	0.87	0.89
Transcriptional regulator ISGF3 gamma subunit	0.60	0.55	0.54	0.74
Interferon-induced 17 kDa protein	0.52	0.15	0.56	0.66
Interferon-inducible protein (p78)	0.42	0.57	0.73	0.57
Epiregulin	0.41	0.51	0.83	0.41
Integrin beta-1	0.41	0.31	0.46	0.48
Lipocalin 2	0.40	0.62	0.75	0.87
Cadherin 6	0.35	0.89	0.63	0.25
Nicotinamide *N*-methyltransferase (NNMT)	0.34	0.35	0.63	0.89
TNF receptor superfamily, member 11b	0.33	0.42	0.67	0.75
Protein kinase C, iota	0.32	0.54	0.63	0.78
Interferon-induced protein 1–8D	0.28	−0.12	−0.03	0.85
Antileukoprotease	0.23	0.81	0.61	0.90
S100 calcium binding protein A2	0.22	0.47	0.36	0.88
Endothelin 1	0.21	0.45	0.42	0.76
Interleukin 1	0.17	0.44	0.66	0.12
Endothelial domain protein 1 (EPAS1)	0.16	0.29	0.61	0.58
Thymosin	0.15	0.54	0.56	0.93
Ceramide glucosyltransferase	0.15	0.31	0.34	0.83
Matrix metalloproteinase 7	0.02	0.39	0.46	0.80
Interleukin 8	−0.03	0.47	0.47	0.86
Interleukin 6	−0.07	0.18	0.29	0.72
S100 calcium binding protein A10	−0.09	0.63	0.15	0.84

a Spearman's rank test was used to calculate correlation coefficients.

**Table 6:** Sensitivity of individual STAT1 clones to various chemotherapeutic drugs

**Stat1 clone**	**Stat1 expression**	**Cisplatin IC_50_ (*μ*M)^a^**	**AMD473 IC_50_ (*μ*M)**	**Oxaliplatin IC_50_ (*μ*M)**
Parental	−	0.23±0.05 (1.0)	2.4±0.5 (1.0)	0.038±0.007 (1.0)
1	+	0.74±0.71 (3.2)	9.3±9.8 (4.0)	0.044±0.009 (1.2)
2	+++	0.95±0.18 (4.1)*	11.8±5.7 (5.0)*	0.065±0.016 (1.7)
3	−	0.24±0.07 (1.1)	3.2±1.6 (1.3)	0.034±0.003 (0.9)
4	−	0.23±0.04 (1.0)	5.4±5.7 (2.3)	0.043±0.011 (1.1)
5	−	0.36±0.05 (1.5)	3.2±1.0 (1.3)	0.026±0.003 (0.8)

a The standard deviation is provided and the relative resistance to parental A2780 cells is shown in parentheses.

* Clones that are statistically different based on a *t*-test with *P*⩽0.05.
